# Establishment of mountain birch (*Betula pubescens* ssp. *tortuosa*) on a glacial outwash plain: Spatial patterns and decadal processes

**DOI:** 10.1002/ece3.9430

**Published:** 2022-10-27

**Authors:** Guðrún Óskarsdóttir, Thóra Ellen Thórhallsdóttir, Anna Helga Jónsdóttir, Hulda Margrét Birkisdóttir, Kristín Svavarsdóttir

**Affiliations:** ^1^ Faculty of Life and Environmental Sciences University of Iceland Reykjavík Iceland; ^2^ Faculty of Physical Sciences University of Iceland Reykjavík Iceland; ^3^ The Soil Conservation Service of Iceland Hella Iceland

**Keywords:** *Betula pubescens ssp. tortuosa*, early successional outwash plain, long‐distance dispersal, mountain birch, priority effects, spatio‐temporal patterns

## Abstract

Most of the Earth's surface has now been modified by humans. In many countries, natural and semi‐natural ecosystems mostly occur as islands, isolated by land converted for agriculture and a variety of other land‐uses. In this fragmented state, long‐distance dispersal may be the only option for species to adapt their ranges in response to changing climate. The order of arrival of species may leave a lasting imprint on community assembly. Although mostly studied at and above the species level, such priority effects also apply at the intraspecific level. We suggest that this may be particularly important in subarctic and arctic ecosystems. Mountain birch (*Betula pubescens* ssp. *tortuosa*) is characterized by great intraspecific variation. We explored spatio‐temporal patterns of the first two mountain birch generations on a homogeneous, early successional glacial outwash plain in SE Iceland that was the recipient of spatially extensive long‐distance dispersal ca. 30 years ago. We evaluated the decadal progress of the young population by remeasuring in 2018, tree density and growth form, plant size, and reproductive effort on 30 transects (150 m^2^) established in 2008 at four sites on the plain and two adjacent sites ca. 10 km away. All measured variables showed positive increases, but contrary to our predictions of converging dynamics among sites, they had significantly diverged. Thus, two of the sites (only 500 m apart) could not be distinguished in 2008, but by 2018, one of them had much faster growth rates than the other, a higher growth form index reflecting more upright tree stature, greater reproductive effort, and much greater second‐generation seedling recruitment. We discuss two hypotheses that may explain the diverging dynamics, site‐scale environmental heterogeneity, and legacies of intraspecific priority effects.

## Graphical abstract

We explored the spatio‐temporal dynamics of the first two generations of mountain birch (*Betula pubescens* ssp. *tortuosa*) on four sites on an outwash plain in SE Iceland. Contrary to predictions of converging dynamics, sites showed significant divergence from 2008 to 2018, and we discuss two hypotheses that may explain this, site‐scale environmental heterogeneity and legacies of intraspecific priority effects. The massive long‐distance (≥10 km) dispersal, spatially extensive colonization (>35 km^2^ area from ca. 1990–2016), and high, although spatially variable, recruitment of the second generation, all illustrate the mountain birch's ability to rapidly adjust its range in a shifting environment.

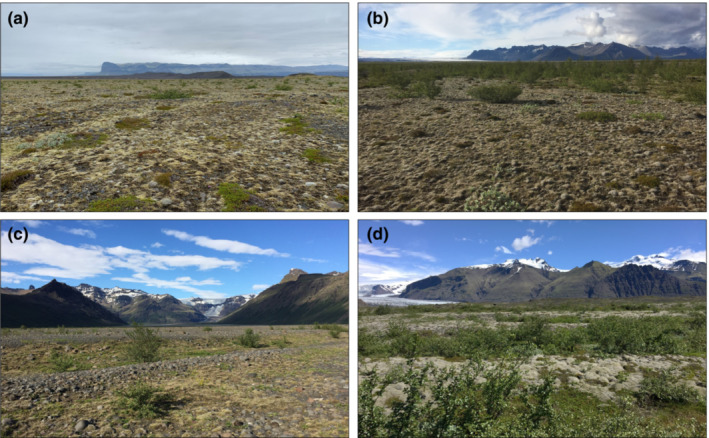

## 1 INTRODUCTION

One consequence of global climate change will be a shift in the distributions of plant populations (Hamann et al., 2021; Körner & Paulsen, 2004). Alpine populations are already shifting to higher elevations, and arctic and subarctic populations are moving polewards (Parmesan & Yohe, 2003). Changes in species distributions may be continuous, with populations contracting or expanding from an existing margin. Alternatively, discrete new populations may establish through long‐distance dispersal (LDD) (Doxford & Freckleton, 2012; Hargreaves & Eckert, 2014). Anthropogenic activities have resulted in extensive habitat loss or degradation, leaving natural ecosystems as isolated islands (Haddad et al., 2015). Although LDD is considered a rare event (Weduwen & Ruxton, 2019), due to today's fragmented state of natural habitats, it may be the only means for many species to reach a suitable habitat.

Plants have little control over the spatial dispersion of their offspring and in most cases, seed dispersal is highly stochastic (Fenner & Thompson, 2005). The successful establishment of a plant population following LDD can be envisaged as having passed through a series of environmental filters (HilleRisLambers et al., 2012). Abiotic filters include climate, microtopography, soil nutrients, and water regime (Harper, 1977; Lett & Dorrepaal, 2018; Pinto et al., 2016). Among the myriad of biotic factors are established plants that can act as competitors, inhibitors, or facilitators (Aradóttir, 2004; Lett et al., 2017; Nystuen et al., 2019), organisms that limit the growth of the new population, e.g., herbivores (Speed et al., 2010; Thórsson, 2008), or symbionts that are crucial for successful establishment, e.g., mycorrhizae (Kokkoris et al., 2020). Each of the above will impose its own scale and degree of patchiness, but the final spatial configurations will determine whether the new species establishes in small discrete patches or as a large, spatially continuous population. The fate of the early colonizers and their offspring, i.e., the first locally recruited generation, will be determined by various spatial and temporal patterns and processes, including the highly stochastic peculiarities of the match or mismatch between the incoming seed rain and the constellation of safe sites (Aradóttir & Halldórsson, 2018) and the genetic constitution of the founder population (Burton et al., 2010; Hargreaves & Eckert, 2014).

The concept of priority effects refers to the legacy or historical contingency that the order of arrival and composition of early species imposes on the structure and function of biological communities (Chase, 2003; Fukami, 2015). The impact of a new arrival will depend on arrival time and the structure and species composition of the receiving ecosystem, e.g., on the suite of functional traits already represented (Körner et al., 2007; Weidlich et al., 2020). For example, a tree species establishing in an early successional, sub‐arctic community consisting of low stature herbs and shrubs will change structural dimensions with its tall persistent woody build, affect microclimate with increased retention of winter snow (Helmutsdóttir, 2022) and altered light regime (D'Odorico et al., 2013), affect soil processes through increased litter deposition and enhanced microbial activity (Jonczak et al., 2020; McElhinny et al., 2010), and attract both vertebrate and invertebrate animals (Kittipalawattanapol et al., 2021; Quinn et al., 2021). The arrival of such an ecosystem engineer will have profound consequences at the ecosystem level and steer the community's successional pathways (Mitchell et al., 2007).

Where there is significant intraspecific structural or functional variation, priority effects may also operate at the population level (Faillace et al., 2022; Jung et al., 2010). We suggest that this may be particularly important in subarctic and arctic ecosystems that have low species richness but harbor ecologically important but sometimes cryptic variation at the subspecies level (Brochmann & Brysting, 2008; Dobbert et al., 2021; Grundt et al., 2006). The spatial configuration and genetic composition of the founder population and first locally recruited generation may thus leave a long‐term legacy, i.e., shape the spatial dynamics of the community for a long time (García‐Girón et al., 2022), but the strength of priority effects may depend on environmental heterogeneity (Tucker & Fukami, 2014).

Mountain birch (*Betula pubescens* subsp. *tortuosa*) displays great variation in growth form, ranging from polycormic decumbent shrubs to monocormic upright trees. We studied the early dynamics following a sudden, large‐scale establishment of mountain birch through LDD onto a sparsely vegetated glacial outwash plain in sub‐arctic Iceland. We report on decadal‐scale spatio‐temporal patterns of density, growth, fecundity, and first local seedling recruitment of the young population, and compare it to two neighboring mountain birch sites. Specifically, we explored whether early demographics of the first generation gave insights into later emerging patterns. Our predictions were that the population characteristics would converge across the flat and apparently homogeneous outwash plain.

## 2 METHODS

### 2.1 Study species

*Betula pubescens* Ehrh. is known through much of its natural range as an early successional forest species (Portsmuth & Niinemets, 2007). However, towards the northern limits of its distribution, it is the dominant tree in stable and regionally important ecosystems (Atkinson, 1992). Its wide habitat tolerance, rapid early growth, and precocious reproductive maturity make *B. pubescens* a highly effective colonizer (Jonczak et al., 2020). In Scotland, for example, it is regarded as a top‐down ecosystem engineer, shaping the community both above‐ and below‐ground (Mitchell et al., 2007). Colonization by *B. pubescens* can have substantial and long‐lasting effects on soil, changing its nutrient supply, pH, and fungal community (Mitchell et al., 2010). Mountain birch (*B. pubescens* ssp. *tortuosa*) is a subspecies of *B. pubescens* native to Fennoscandia (Panarctic Flora, n.d.), generally found towards the altitudinal and latitudinal limits of the species (Atkinson, 1992; Holm, 1994). All native birch in Iceland is regarded as belonging to this subspecies (Kristinsson et al., 2018).

During early primary succession, light is generally abundant, and the shade‐intolerant *B. pubescens* (Portsmuth & Niinemets, 2007) can establish due to its ability to grow in nutrient poor soils (Atkinson, 1992). However, surface instability may limit establishment in barren areas, and insufficient seed rain precludes colonization of areas far away from seed sources (Aradóttir & Halldórsson, 2018). In Iceland, these limitations apply over a regionally extensive land, for example, on large glacial outwash plains.

### 2.2 Study area

**FIGURE 1:**
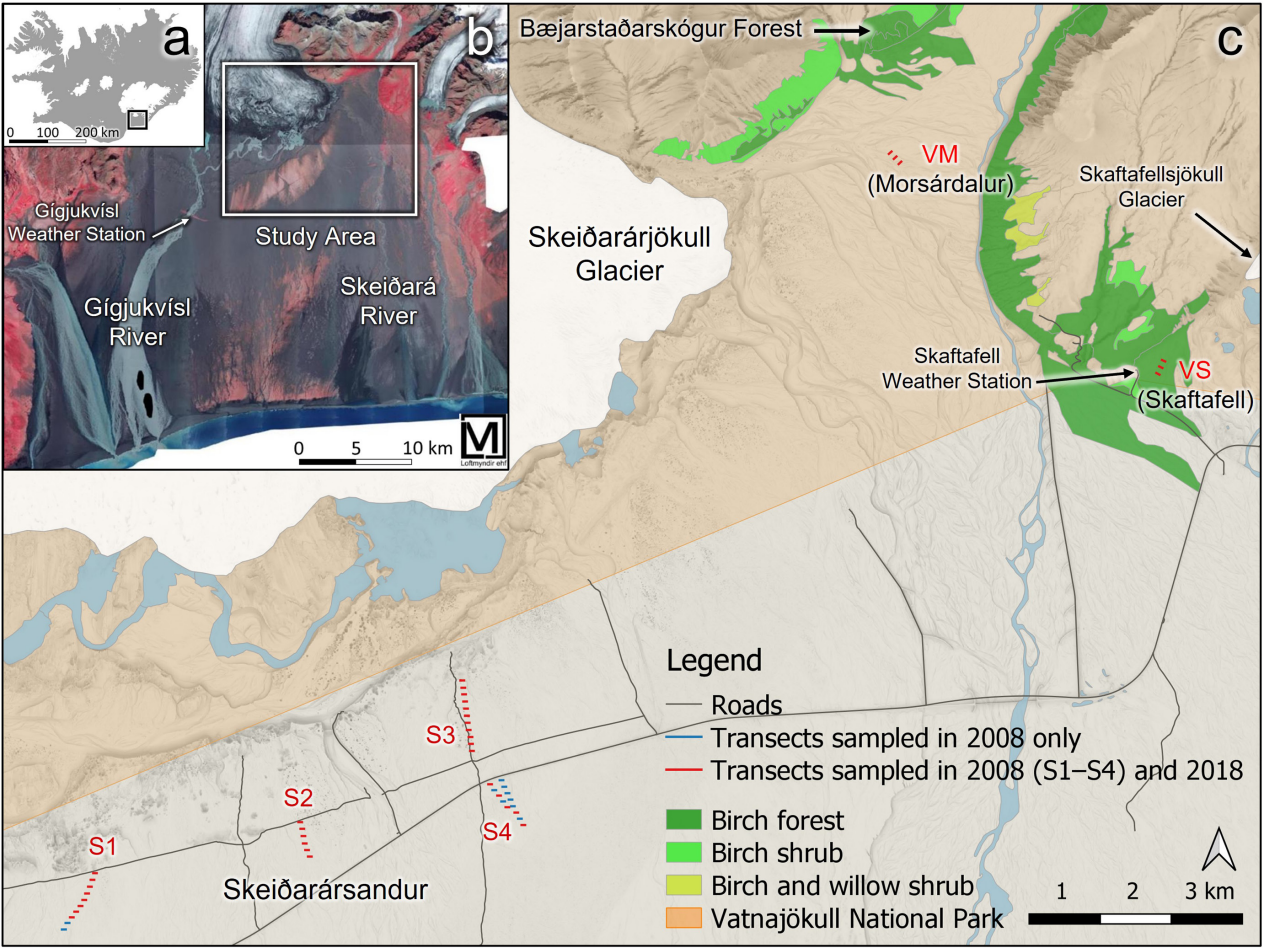
Location of Skeiðarársandur (SKS) in SE Iceland (a), the study area within SKS and Vatnajökull National Park (VNP) on an infrared aerial photo (vegetation in red) (b), and the sites/transects on SKS (S1–S4) and in VNP, Morsárdalur (VM) and Skaftafell (VS) (c). Site names are in red font. Map database: National Land Survey of Iceland (2020). Aerial photos: Loftmyndir ehf. (n.d.). Mountain birch map data: Icelandic Institute of Natural History (2019). Geographic information system: QGIS (QGIS Development Team, 2020).

The main research area is within the 1000 km^2^ Skeiðarársandur (SKS) glacial outwash plain (63°58′N, 17°12′W, Figure 1a). Since the 14th century, at least, SKS has regularly received outburst floods, leaving it extremely barren by the late Little Ice Age. After the mid‐20th century, the disturbance regime had changed, allowing the establishment of early successional vegetation (discussion in Thórhallsdóttir & Svavarsdóttir, 2022). Still, 70% of the central part of the plain between the rivers Gígjukvísl and Skeiðará had <10% vegetation cover in 2002 (Kofler, 2004), and most of SKS remains sparsely vegetated (Figure 1b). In the upper zone of the plain (60–110 m a.s.l.), the substrate is coarser and more stable than in the sandier part seawards. Within that upper zone, mountain birch has established across at least 35 km^2^ (V. P. Madrigal et al., unpublished data), despite the nearest seed source being >10 km away. Age distributions based on dendrochronology indicate that mountain birch colonized the area around 1990 (H. M. Birkisdóttir et al., unpublished data; Hiedl et al., 2009; Marteinsdóttir et al., 2007).

**FIGURE 2:**
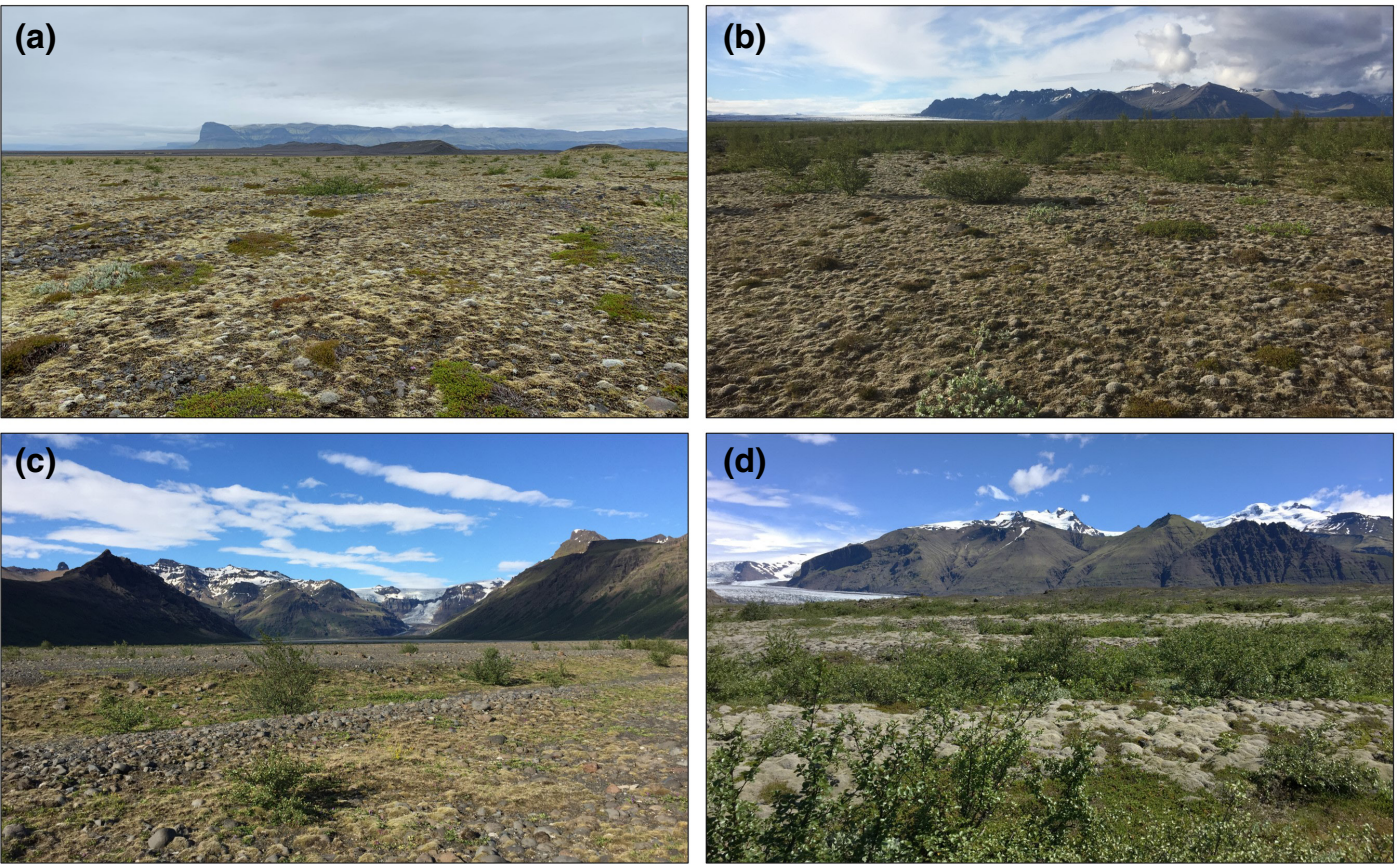
Examples of the study sites representing the range of conditions on Skeiðarársandur (a: S1, b: S4) and in Vatnajökull National Park (c: VM, d: VS). The maximum distance between sites is 16 km (S1–VS).

Ecosystem development on the plain has been studied since shortly after mountain birch colonization and establishment (Hiedl et al., 2009; Kofler, 2004; Marteinsdóttir et al., 2007, 2010, 2013, 2018; Thórhallsdóttir & Svavarsdóttir, 2022). In 2008, Hiedl et al. (2009) gathered extensive data on the mountain birch population, summarizing its demographics at four sites in the west (S1), central (S2), northeast (S3), and southeast (S4) parts of the mountain birch area (Figure 1c). By then, the largest trees had reached reproductive maturity, but despite an extensive survey, no first‐year seedlings were found (Hiedl et al., 2009). At S1 and S2, mountain birch plants were small and sparse, but at S3 and S4, trees were denser and larger (Figure 2). Despite differences in mountain birch density, a vegetation survey conducted in 2018 found similar vegetation composition at all sites (G. Óskarsdóttir et al., unpublished data), with the sward layer dominated by *Racomitrium lanuginosum* and *R. ericoides* (80%, 77%, and 66% average combined cover at S1, S3, and S4, respectively). Other common species included shrubs and dwarf shrubs (*Empetrum nigrum, Salix lanata, S. herbacea*, *Calluna vulgaris*), graminoids (*Juncus trifidus, Festuca richardsonii*, *F. vivipara*), and *Stereocaulon* lichens (Figure 2). For the past decades, SKS has been grazed in summer by around 200 ewes (Thórhallsdóttir & Svavarsdóttir, 2022).

In 2018, two sites were selected within Vatnajökull National Park (VNP) to compare the SKS population to its nearest neighboring mountain birch stands. Site VM is in Morsárdalur valley (Figure 1c), where mountain birch established around 1990 (Thórhallsdóttir & Svavarsdóttir, personal observations), but tree density is still low (Figure 2). Site VS is on the proglacial area in front of Skaftafellsjökull, near Skaftafell weather station (Figure 1c), where mountain birch had begun to establish by the early 1960s (Persson, 1964). While most of the young trees at VM and many on SKS have a largely upright and tree‐like growth form, the mountain birch at VS is generally multi‐stemmed and more procumbent (Figure 2). VM is largely sparsely vegetated, but the ground at VS is mostly covered with *Racomitrium* moss and various dwarf shrubs (Figure 2).

Southeast Iceland has a maritime climate with high precipitation. At Skaftafell weather station (86 m a.s.l.; Figure 1c), mean January and July temperatures are 0.9 and 10.9°C, respectively, the mean annual temperature is 5.2°C, and the mean annual precipitation is around 1650 mm (1996–2019, unpublished data from the Icelandic Meteorological Office, www.vedur.is). Six years of data (2014–2019) are available for a temperature station on SKS itself, 5–11 km from S1–S4 (Gígjukvísl, 58 m a.s.l., unpublished data from the Icelandic Meteorological Office, www.vedur.is; Figure 1b). Mean June–August temperatures were comparable for the two stations (10.3°C vs. 10.4°C for the same years in Skaftafell), but it is likely that Skaftafell has higher precipitation, due to proximity to high mountains, and generally lower windspeeds than Gígjukvísl (The Technical University of Denmark, 2021). Prevailing winds are from the north–east (Icelandic Meteorological Office, n.d.).

### 2.3 Sampling design

To assess temporal changes in mountain birch demographics on SKS, we built on the 2008 survey of Hiedl et al. (2009) where at each of the four sites on SKS (S1–S4, Figure 1c), 150 m^2^ (3 × 50 m) transects were established at 100 m intervals southwards, until the whole north–south spread of mountain birch had been covered, or to a maximum distance of 1000 m from the first transect. At S4, two adjacent N–S series of transects were established to increase the sample size. In total, 40 transects were established in 2008, of which 30 were resampled in 2018 (Figure 1c). The high plant density at S4 made complete resampling too time consuming, and a subset of five transects was selected, three from the western series and two from the eastern one, extending across the whole sampling area from north to south. At S1, GPS coordinates for the two southernmost transects of 2008 were missing, so only the remaining eight transects were resampled.

Both VNP sites comprised three 150 m^2^ (3 × 50 m) transects, oriented perpendicular to dry riverbanks (Figure 1c). In total, 36 transects were sampled in 2018, covering 5400 m^2^, thereof 4500 m^2^ within SKS.

### 2.4 Data sampling

In 2008, maximum plant height, length of the longest shoot, and the number of female catkins were recorded for all mountain birch plants within each transect. Since male catkins were not counted, *catkins* hereafter refers to female ones. We use *plant size* and *height* when referring to the length of its longest shoot and greatest height above ground, respectively (see Section 2.5 for further details). We use *trees* when referring to the largest plant category (≥20 cm) in our sample. For the remaining plants in the 2018 resampling, plant size only was measured for plants between 1 and 5 cm (here referred to as *larger seedlings, or L‐seedlings*). Due to their large numbers, we counted but did not measure ≤1 cm plants (here referred to as *smaller seedlings, or S‐seedlings*), and the size of all was assigned 1 cm. No first‐year seedlings (plants with cotyledons) were quantified.

At the three northernmost transects at S4, the number of S‐seedlings was so great that we counted a subsample in four 0.25 m^2^ quadrats, placed at 33 cm intervals (widthwise) at every other metre (lengthwise) along each transect (*n* = 100 per transect). Estimated total number of S‐seedlings was then extrapolated for each of those transects. Due to VM's small sample size of trees (*n* = 3, mean size = 126 cm), size and height of 40 additional randomly chosen trees (mean size = 110 cm) were measured in June 2019 and added to the 2018 dataset.

### 2.5 Data analyses

For each transect, total density was calculated as the number of all individuals divided by area. Similarly, tree density was calculated using the number of individuals ≥20 cm, flowering adult density by using individuals with catkins, and finally, catkin density by using the total number of catkins. Plant height and the length of its longest shoot are interchangeable for upright trees, but since some plants in our study were prostrate or grew at an angle, we used the latter as a main measure of size. For the same reason, the ratio between plant height and shoot length was used as an index to study spatio‐temporal variation in growth form.

We assessed the decadal‐scale progress of the SKS population by comparing its status in 2008 and 2018 and exploring between‐site variation. We also investigated between‐site variation for 2008 separately on one hand, and for 2018 on the other, including the two VNP sites in the latter case. The following response variables were examined: density of all plants, trees, flowering adults, and catkins, presence and abundance of catkins per plant (between‐site variation in 2008 not studied, and S‐/L‐seedlings excluded due to their very high numbers and low likelihood of persistence in the population), plant size (only trees included, due to increased number of young individuals between years), and plant growth form index (S‐/L‐seedlings excluded due to missing height data). The additional trees measured at VM in 2019 were only used in analyses of plant size and growth form (Sections 3.3 and 3.4).

All data handling and analyses were conducted in *R* v3.6.2. (R Core Team, 2019). Graphs were produced using the package *ggplot2* v3.2.1 (Wickham, 2016). Negative binomial (NB) regressions were fitted with the *MASS* package v7.3‐57 (Venables & Ripley, 2002). Negative binomial mixed models (NBMM) and hurdle/zero‐altered negative binomial mixed models (hurdle models) were fitted with the package *glmmTMB* v1.1.2.3 (Brooks et al., 2017). Linear mixed models (LMM) were fitted with the package *nlme* v3.1‐142 (Pinheiro et al., 2019). Linear models (LM) were fitted with the package *stats* (R Core Team, 2019). Likelihood ratio tests (LRT) were performed using the package *lmtest* v0.9‐38 (Zeileis & Hothorn, 2002). Analysis of variance (ANOVA) type II tests were conducted with the *car* package (Fox & Weisberg, 2019). Estimated marginal means (EMMs) were calculated and plotted using the package *emmeans* v1.4.5 (Lenth, 2020). Summary of all models is presented in Table S1.

#### 2.5.1 Density and catkin production

To explore spatio‐temporal variations in mountain birch density, the density of trees, flowering adults, and their catkins, we used NBMM (with log link) with *year*, *site*, and their interaction as explanatory variables and *transect* (nested within sites) as a random effect (Table S1). Site differences for each year were explored using NB regressions. Since all transects were the same size, we used plant/catkin numbers instead of their calculated density for the NBMM and NB regressions. We used EMMs for temporal and spatial pairwise comparisons. For all models, sample sizes equalled the number of transects (S1 = 8, S2 = 6, S3 = 11, S4 = 5, VM = 3, VS = 3).

#### 2.5.2 Presence and abundance of catkins

Hurdle model (truncated negative binomial, with log link) with *transect* (nested within sites) as a random effect was used to explore how the presence and abundance of catkins related to plant size and differed between sites and years (Table S1). A hurdle model is a two‐part model, consisting of a zero‐part and a zero‐truncated count‐part. In the zero‐part, the probability of a plant having catkins was estimated, using logistic regression. In the count‐part, only flowering plants were included, and the number of catkins was estimated, using NB regression (Zuur et al., 2009).

Two hurdle models were built. The first one included only the SKS data from 2008 and 2018, aiming to identify whether the presence and abundance of catkins in relation to plant size had changed as the SKS population grew older, using *site, plant size, year*, and interaction between the two latter variables as fixed effects (Table S1). The second model included the 2018 data from SKS and VS (at VM, catkins occurred only on one of the sampled plants, thus the site was excluded), aiming to assess spatial variation in the presence and abundance of catkins in relation to plant size, using *site, plant size*, and their interaction as fixed effects (Table S1). In both models, for both model parts, we used backwards elimination for model reduction, with LRT (*α* < 0.05; Zuur et al., 2009). Sample sizes equalled the number of mountain birch plants, excluding S‐ and L‐seedlings (2008: S1 = 29, S2 = 17, S3 = 157, S4 = 52; 2018: S1 = 65, S2 = 25, S3 = 162, S4 = 205, VS = 393).

#### 2.5.3 Tree size

Spatio‐temporal variation in tree size within SKS was studied using LMM with *transect* (nested within sites) as random effect and *year, site*, and their interaction as fixed effects (Table S1). The model had separate variance components for each year to account for different variances. Differences in tree size between SKS sites in 2008, on one hand, and all sites in 2018, on the other, were analyzed using comparable LMMs, but for those two models, the only fixed effect was *site*. For all LMMs, *tree size* values were log‐transformed to meet model assumptions. We used EMMs for temporal and spatial pairwise comparisons. Sample sizes equalled the number of trees (2008: S1 = 13, S2 = 10, S3 = 115, S4 = 37; 2018: S1 = 40, S2 = 11, S3 = 128, S4 = 80, VM = 43, VS = 226).

The spatial differences in local recruitment may confound comparisons of tree growth among SKS sites and between years. For this reason, and to assess potential canopy height, we studied a sample of the 20 largest trees at each site separately. To study spatio‐temporal patterns in the growth of those trees, we used LM with *year, site*, and their interaction as explanatory variables (Table S1). Spatial patterns on SKS in 2008, on one hand, and at all sites in 2018, on the other, were also studied using LM, including the variable *site*. The dependent variables were log‐transformed if needed to meet model assumptions (see Table S1). We used EMMs for temporal and spatial pairwise comparisons. The sample size was 20 for each site.

#### 2.5.4 Plant growth form

For growth form comparisons on SKS in 2008 and 2018, we used LMM on *plant height/size ratio* with *year, site*, and their interaction as fixed effects and *transect* (nested within sites) as a random effect (Table S1). Differences in the plant growth form index between sites on SKS in 2008, on one hand, and on SKS and VNP in 2018, on the other, were also explored using LMMs, but for those models, the only fixed effect was *site*. We used EMMs for temporal and spatial pairwise comparisons. Sample sizes equalled the number of mountain birch plants, excluding S‐ and L‐seedlings (2008: S1 = 29, S2 = 17, S3 = 157, S4 = 52; 2018: S1 = 65, S2 = 25, S3 = 162, S4 = 205, VS = 393).

## 3 RESULTS

### 3.1 Density and catkin production

Catkin production of the Skeiðarársandur (SKS) mountain birch greatly increased between 2008 and 2018, and all density‐related variables had elevated values (Tables 1 and 2; Table S1). Significant between‐site differences were found in all among‐year comparisons (Tables 1 and 2). Densities were lower at the westernmost (S1 and S2) than at the easternmost sites (S3 and S4). For the most part, plant and catkin densities were very different between the two Vatnajökull National Park (VNP) sites, and while VM values were similar to values recorded for S1 and S2 in 2018, VS values were more similar to those recorded for S3 and S4 (Table 2).

**TABLE 1:** ANOVA type II test results for NBMM and NB regressions of the number of mountain birch plants (excluding first‐year seedlings), trees (≥20 cm), flowering adults, and their catkins at all Skeiðarársandur (SKS) sites in 2008 and 2018 and both Vatnajökull National Park (VNP) sites in 2018.

Data	Factor	*df*	All plants		Trees		Flowering		Catkins	
			*χ* ^2^	*p*	*χ* ^*2*^	*p*	*χ* ^*2*^	*p*	*χ* ^*2*^	*p*
SKS 2008 and 2018	Year	1	123.2	**<.001**	9.4	**.002**	36.8	**<.001**	23.6	**<.001**
	Site	3	102.8	**<.001**	32.9	**<.001**	26.7	**<.001**	41.9	**<.001**
	Year: Site	3	168.8	**<.001**	8.4	**.038**	4.5	.208	9.2	**.027**
SKS 2008	Site	3	26.3	**<.001**	28.8	**<.001**	32.8	**<.001**	33.8	**<.001**
SKS and VNP 2018	Site	5	294.8	**<.001**	115.3	**<.001**	83.8	**<.001**	28.4	**<.001**

*Note*: Significant values are in bold (*p* < .05). Sample sizes equalled the number of transects (S1 = 8, S2 = 6, S3 = 11, S4 = 5, VM = 3, VS = 3).

Abbreviations: df, degrees of freedom; *χ* ^2^, chi‐square value; *p*, *p*‐value.

**TABLE 2:** Sampling effort and density (means ± standard errors) of all mountain birch plants (excluding first‐year seedlings), trees (≥20 cm), flowering adults, and their catkins on Skeiðarársandur (S1–S4) in 2008 and 2018 and in Vatnajökull National Park (VM and VS) in 2018. Lowercase and uppercase letters in superscript denote significant differences between sites in 2008 and 2018, respectively (EMMs, *α* < 0.05).

Year	Site	Sampled area (m^2^)	Density (plants/m^2^)			
			All plants	Trees	Flowering	Catkins
2008	S1	1200	0.033 ± 0.005^ab^	0.011 ± 0.003^a^	0.001 ± 0.001^ab^	0.002 ± 0.002^a^
	S2	900	0.021 ± 0.011^a^	0.011 ± 0.009^a^	0.001 ± 0.001^ab^	0.013 ± 0.013^a^
	S3	1650	0.125 ± 0.028^c^	0.070 ± 0.015^b^	0.016 ± 0.004^c^	0.898 ± 0.401^b^
	S4	750	0.081 ± 0.019^bc^	0.049 ± 0.013^b^	0.012 ± 0.004^bc^	1.315 ± 0.552^b^
2018	S1	1200	0.085 ± 0.017^A^	0.033 ± 0.006^AB^	0.014 ± 0.004^A^	0.248 ± 0.120^AB^
	S2	900	0.072 ± 0.014^A^	0.012 ± 0.006^A^	0.008 ± 0.004^A^	0.211 ± 0.194^BC^
	S3	1650	0.153 ± 0.031^AB^	0.078 ± 0.015^BC^	0.043 ± 0.008^BC^	2.149 ± 0.711^CD^
	S4	750	9.493 ± 3.550^D^	0.107 ± 0.036^C^	0.056 ± 0.022^C^	11.104 ± 5.158^D^
	VM	450	0.342 ± 0.173^BC^	0.007 ± 0.007^A^	0.002 ± 0.002^AB^	0.138 ± 0.138^ABC^
	VS	450	1.171 ± 0.517^C^	0.502 ± 0.113^D^	0.180 ± 0.000^D^	2.353 ± 0.268^BCD^

*Note*: Sample sizes equalled the number of transects (S1 = 8, S2 = 6, S3 = 11, S4 = 5, VM = 3, VS = 3).

### 3.2 Presence and abundance of catkins

The hurdle model for the presence and abundance of catkins on SKS in 2008 and 2018 showed no significant interaction between *year* and *plant size* (Table 3). Therefore, the predicted probability of SKS plants' presence (zero‐part of the model) and abundance (count‐part of the model) of catkins in relation to plant size did not differ temporally, although their presence varied between years and sites. Backward elimination of the full model, as shown in Table 3 (using LRT), resulted in the best subset model, including *plant size* in both parts, as well as *year* and *site* in the zero‐part (Table S3). According to EMMs on that model, the predicted probability of catkin presence was higher at S2/S3 than at S4, and higher in 2018 than in 2008 (Table S4). However, only plant size had significant effects on predicted catkin abundance (Table S3).

**TABLE 3:** ANOVA type II test results for hurdle models of the presence and abundance of catkins at all Skeiðarársandur (SKS) sites in 2008 and 2018, and one Vatnajökull National Park site (VS) in 2018.

Model part	SKS 2008 and 2018				SKS and VS 2018			
	Factor	*df*	*χ* ^2^	*p*	Factor	*df*	*χ* ^2^	*p*
Zero	Year	1	7.6	**.006**	Size	1	180.0	**<.001**
	Size	1	148.5	**<.001**	Site	4	18.0	**.001**
	Site	3	13.4	**.004**	Size:Site	4	5.2	.267
	Year:Size	1	1.4	.236				
Count	Year	1	0.3	.578	Size	1	32.4	**<.001**
	Size	1	25.1	**<.001**	Site	4	23.9	**<.001**
	Site	3	7.5	.059	Size:Site	4	2.0	.745
	Year:Size	1	2.3	.133				

*Note*: Results from full models are shown. Significant values are in bold (*p* < .05). Sample sizes equalled the number of mountain birch plants, excluding S‐ and L‐seedlings (2008: S1 = 29, S2 = 17, S3 = 157, S4 = 52; 2018: S1 = 65, S2 = 25, S3 = 162, S4 = 205, VS = 393).

Abbreviations: *df*, degrees of freedom; *χ* ^*2*^, chi‐square value; *p*, *p*‐value.

**FIGURE 3:**
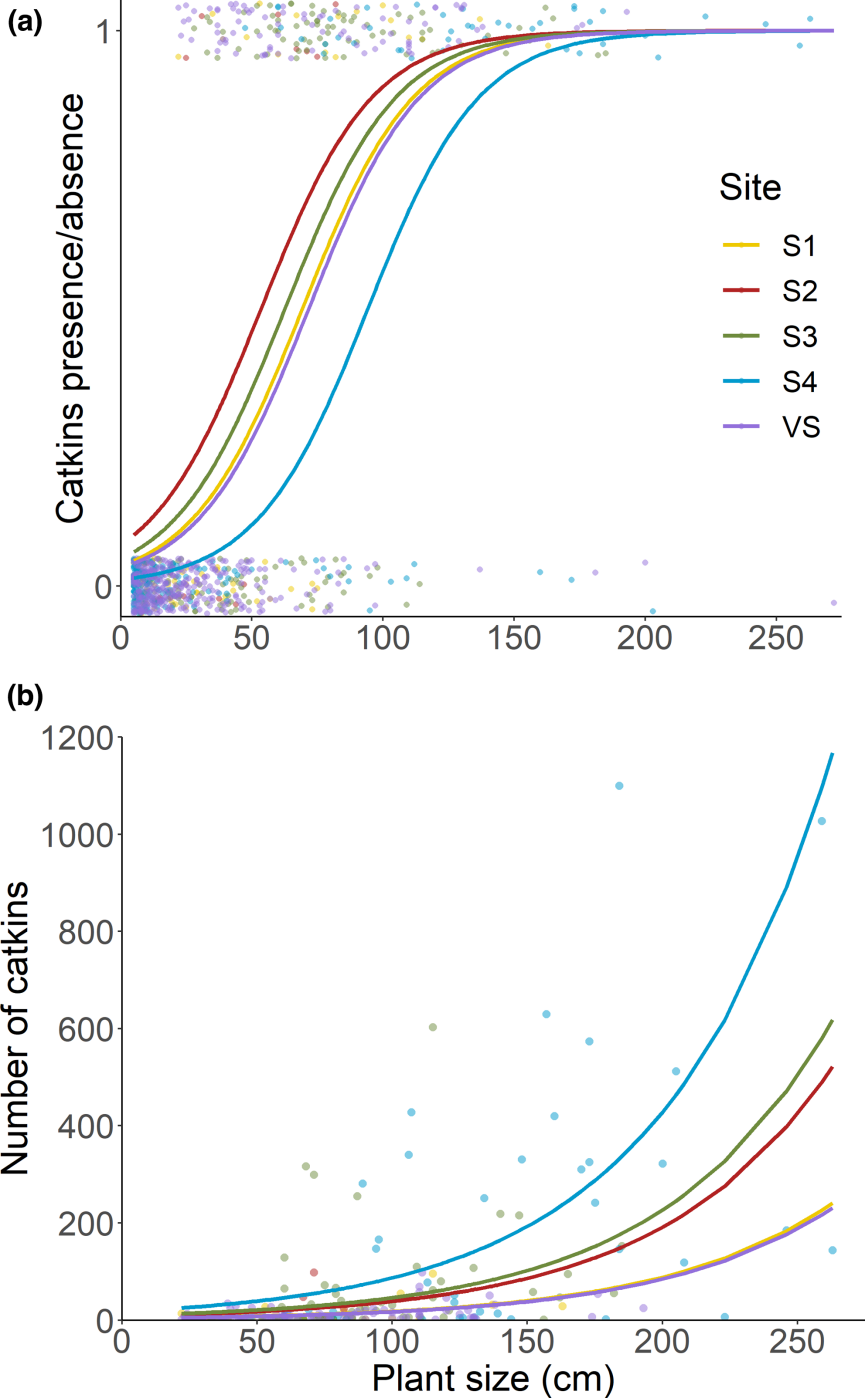
Predicted probability of mountain birch catkin presence (a), and abundance (b) in relation to plant size (length of its longest shoot) for the Skeiðarársandur sites (S1–S4) and one Vatnajökull National Park site (VS) in 2018. Results from the reduced model (see Tables S1 and S3) are shown. Sample sizes equalled the number of mountain birch plants, excluding S‐ and L‐seedlings (2008: S1 = 29, S2 = 17, S3 = 157, S4 = 52; 2018: S1 = 65, S2 = 25, S3 = 162, S4 = 205, VS = 393).

For the spatial variation in 2018, including the VS data, *plant size x site* interaction was not significant (Table 3), and according to LRT, could be dropped from both parts of the model (Table S3). Therefore, in Figure 3, results for the reduced hurdle model (Table S1) on the presence and abundance of catkins on SKS and VS in 2018 are presented, using the variables *plant size* and *site* in both parts. Plant size had highly significant effects in both parts of the model (Table 3). Looking at each part of the model separately, the predicted probability of catkin presence was lower at S4 than at S2, S3, and VS (Figure 3a; Table S4), but of all flowering adults, catkin predicted abundance was highest at S4, with significant difference between S4 and S1/VS on one hand, and S3 and VS on the other (Figure 3b; Table S4).

### 3.3 Tree size

**FIGURE 4:**
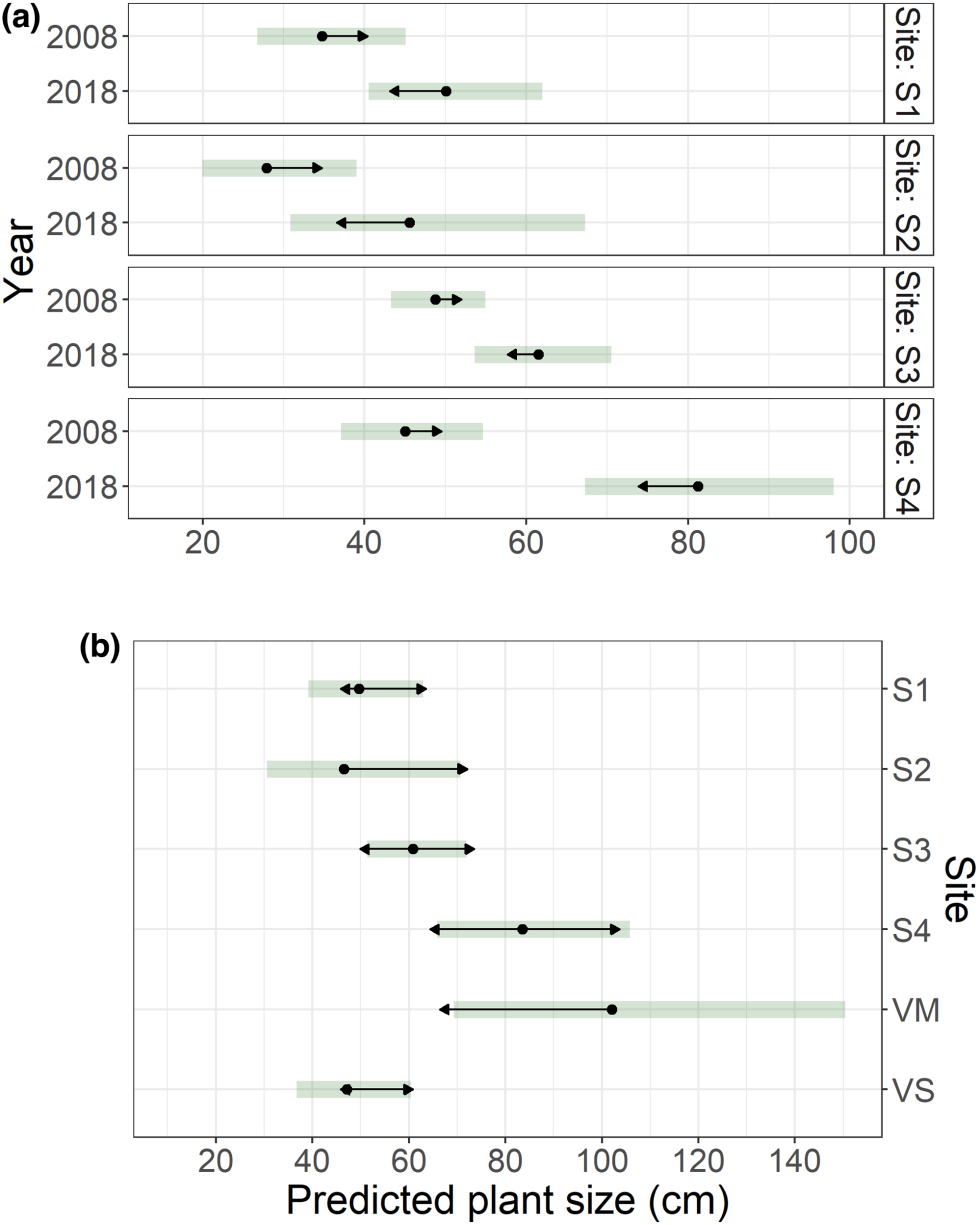
EMMs based on LMMs of mountain birch tree (≥20 cm) size (length of the longest shoot), showing (a) temporal change for each Skeiðarársandur site (S1–S4) between 2008 and 2018, and (b) spatial variation in 2018, including the Vatnajökull National Park sites (VM and VS). The bars show 95% confidence intervals for the EMMs, and the arrows comparisons among them. If an arrow from one mean overlaps an arrow from another group, the difference is not significant (*α* < 0.05). Note that the x‐axes differ between graphs, and a comparison between sites cannot be made using Figure 4a, because arrows are not comparable between them. Sample sizes equalled the number of trees (2008: S1 = 13, S2 = 10, S3 = 115, S4 = 37; 2018: S1 = 40, S2 = 11, S3 = 128, S4 = 80, VM = 43, VS = 226).

The LMM for tree size on SKS in 2008 and 2018 revealed significant effects of fixed factors and their interaction (Table 4), reflecting different growth rates between sites (Figure 4a). LMM on the 2008 data (not shown in figure) revealed significant differences between sites (Table 4), with plants significantly smaller at S2 than at S3 (*z*‐value = −3.442, *p* = .012) and S4 (*z*‐value = −2.813, *p* = .047). In 2018, the difference between sites was also significant (Table 4), with plants on average larger at S4 and VM than at S1 and VS (Figure 4b).

**TABLE 4:** ANOVA type II test results for LMMs of tree (≥20 cm) size (*χ* ^2^) and LMs of the size of the 20 largest trees (*F*) at all Skeiðarársandur (SKS) sites and both Vatnajökull National Park (VNP) sites in 2008 and 2018.

Data	Factor	SKS 2008 and 2018			SKS 2008			SKS and VNP 2018		
		*df*	*χ* ^2^ */F*	*p*	*df*	*χ* ^2^ */F*	*p*	*df*	*χ* ^2^ */F*	*p*
All trees	Year	1	50.3	**<.001**						
	Site	3	20.3	**<.001**	3	16.3	**<.001**	5	24.3	**<.001**
	Year:Site	3	9.7	**.021**						
20 largest trees	Year	1	112.7	**<.001**						
	Site	3	114.8	**<.001**	3	45.1	**<.001**	5	60.8	**<.001**
	Year:Site	3	7.4	**<.001**						

*Note*: Significant values are in bold (*p* < .05). Sample sizes in the LMMs equalled the number of trees (2008: S1 = 13, S2 = 10, S3 = 115, S4 = 37; 2018: S1 = 40, S2 = 11, S3 = 128, S4 = 80, VM = 43, VS = 226).

Abbreviations: *df*, degrees of freedom; *χ* ^*2*^, chi‐square value; *F*, *F*‐value; *p*, *p*‐value.

**FIGURE 5:**
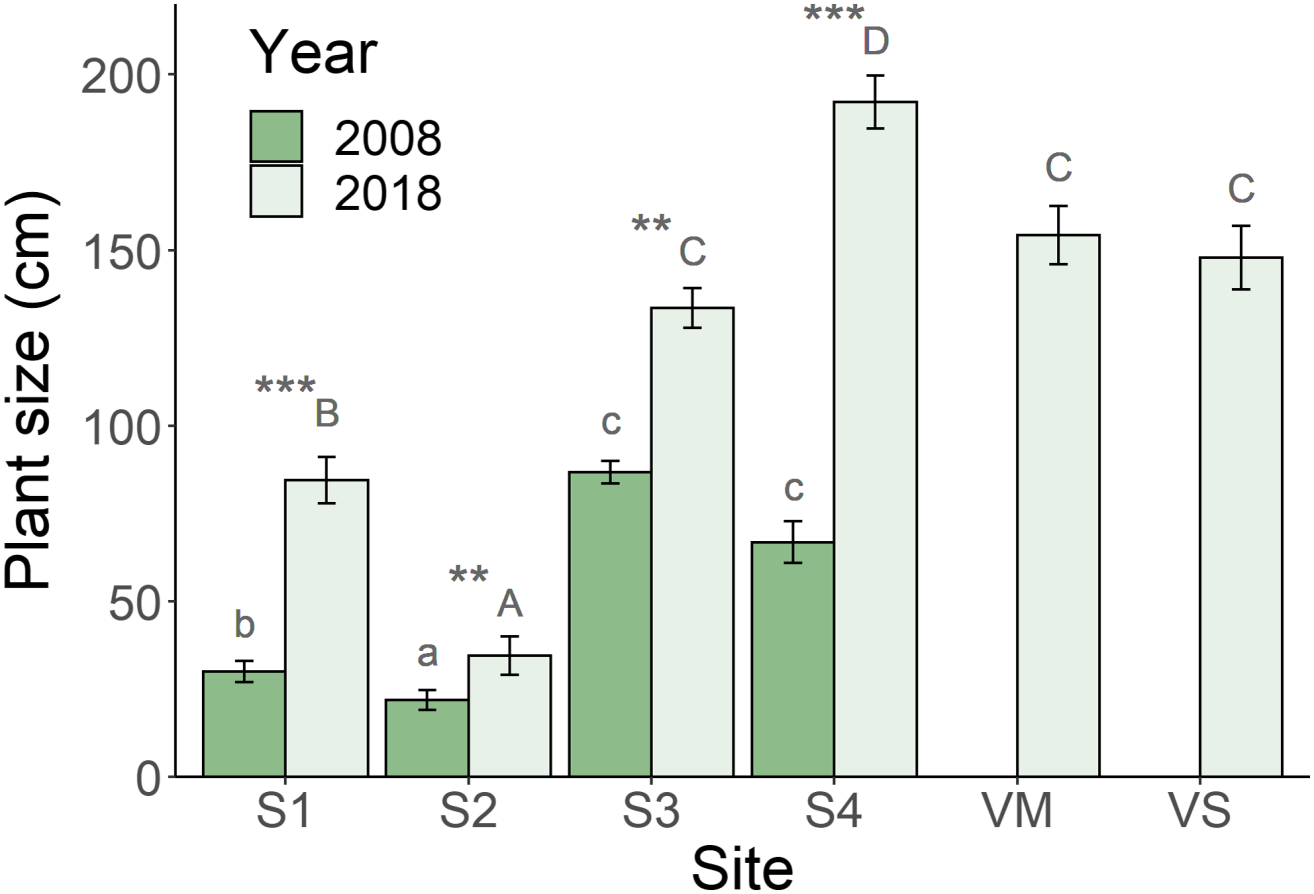
Average size (with standard errors) of the 20 largest mountain birch trees at each site in 2008 and 2018. Lowercase and uppercase letters denote significant differences between sites in 2008 and 2018, respectively, while asterisks denote significant differences between years for each site (EMMs, *α* < 0.05). At S2 in 2008, the total sample size was only 19 plants.

For the 20 largest trees at each site, LM on the SKS data in 2008 and 2018 revealed significant effects of fixed factors and their interaction (Table 4). During the study period, the growth rate of the largest trees thus differed between sites (Figure 5). Between‐site differences were also noticed when studying each year separately (Figure 5; Table 4). In 2018, neither VNP site was significantly different from S3, but a significant difference was found between all other sites (Figure 5).

### 3.4 Plant growth form

**FIGURE 6:**
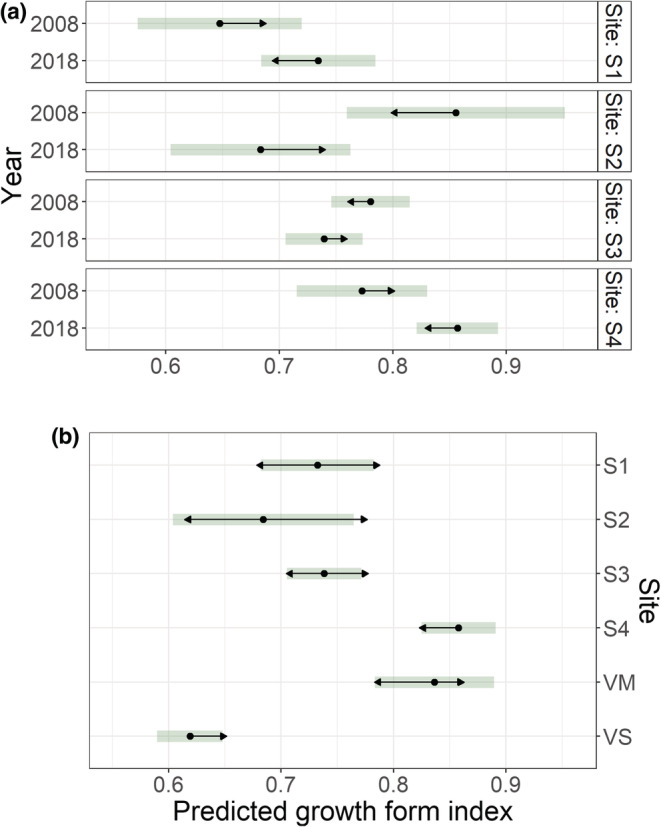
EMMs based on LMMs of mountain birch growth form index (largest shoot height to length ratio), showing (a) temporal change for each Skeiðarársandur site (S1–S4) between 2008 and 2018, and (b) spatial variation in 2018, including the Vatnajökull National Park sites (VM and VS). The bars show 95% confidence intervals for the EMMs, and the arrows comparisons among them. If an arrow from one mean overlaps an arrow from another group, the difference is not significant (*α* < 0.05). Note that a comparison between sites cannot be made using Figure 6a, because arrows are not comparable between them. Sample sizes equalled the number of mountain birch plants, excluding S‐ and L‐seedlings (2008: S1 = 29, S2 = 17, S3 = 157, S4 = 52; 2018: S1 = 65, S2 = 25, S3 = 162, S4 = 205, VM = 67, VS = 393).

Temporal patterns of the plant growth form index varied among SKS sites (Table 5; Figure 6a), and between‐site variation was notable in both years (Table 5). In 2008 (data not shown in figure), index values were lower at S1 than at S2 (*z*‐value = −3.334, *p* = .013) and S3 (*z*‐value = −3.213, *p* = .018). In 2018, however, plants were most upright at S4 and VM, but least upright at site VS, which had significantly lower index values than all other sites, except S2 (Figure 6b).

**TABLE 5:** ANOVA type II test results for LMMs of plant growth form index (largest shoot height to length ratio) at all Skeiðarársandur (SKS) sites in 2008 and 2018 and both Vatnajökull National Park (VNP) sites in 2018.

Factor	SKS 2008 and 2018			SKS 2008			SKS and VNP 2018		
	*df*	*χ* ^2^	*p*	*df*	*χ* ^2^	*p*	*df*	*χ* ^*2*^	*p*
Year	1	0.0	.965						
Site	3	27.4	**<.001**	3	14.1	**.003**	5	141.5	**<.001**
Year:Site	3	25.3	**<.001**						

*Note*: Significant values are in bold (*p* < .05). Sample sizes equalled the number of mountain birch plants, excluding S‐ and L‐seedlings (2008: S1 = 29, S2 = 17, S3 = 157, S4 = 52; 2018: S1 = 65, S2 = 25, S3 = 162, S4 = 205, VM = 67, VS = 393).

Abbreviations: *df*, degrees of freedom; *χ* ^*2*^, chi‐square value; *p*, *p*‐value.

## 4 DISCUSSION

### 4.1 From long‐distance dispersal to self‐sustaining population

For the first generation of mountain birch to establish on the plain, the plants had to pass through several environmental filters, one of which was seed dispersal (HilleRisLambers et al., 2012). Seed rain densities for wind dispersed seeds, such as *Betula pubescens*, decline steeply with distance from the mother plant (Aradóttir, 1991; Fenner & Thompson, 2005), and successful colonization kilometers away is rare (Doxford & Freckleton, 2012; Weduwen & Ruxton, 2019). Preliminary analyses of potential parent populations show that the mountain birch on Skeiðarársandur (S4) is derived from the woodland approximately 10 km northeast of the plain, predominantly from Bæjarstaðarskógur forest (K. P. Magnússon et al., unpublished data). Long‐distance dispersal over roughly 10 km of mostly non‐suitable habitats (barren and unstable sand) was needed for first generation establishment, showing the species' ability to shift its geographical range, even in a fragmented landscape (Hargreaves & Eckert, 2014).

Mountain birch density and catkin production on Skeiðarársandur greatly increased during the study period, although temporal patterns were mostly site‐specific (Table 1, 2 and Table S1). Like most northern hemisphere trees, mountain birch is regarded as a masting species, i.e., it intermittently produces large seed crops with synchrony across extensive geographic regions (Holm, 1994; Koenig & Knops, 2000; Zamorano et al., 2018). Therefore, the patterns in 2008 and 2018 might not be representative for other years. We do not have information on masting in the area, but the increased tree size in the study period (Figures 4a and 5), along with the rising predicted presence and abundance of catkins with plant size (Figure 3), all support a conclusion of greatly increased reproductive effort through the study period, noting that each catkin usually contains around 200 seeds (Holm, 1994). Looking further back, the increase becomes even more pronounced. In 2004, Marteinsdóttir et al. (2007) counted flowering adults and catkins in three areas, approximately corresponding to S1, S3, and S4. Despite a much larger research area (15,800 m^2^), only 10 flowering adults were recorded (3% of the sample), with a total of 106 catkins, or 0.007 catkins/m^2^. Using these data for comparison, catkin density had increased 150‐fold by 2008, to 1.0 catkin/m^2^, and almost 700‐fold by 2018, to 4.7 catkins/m^2^.

In 2004, most individuals in the then roughly 15‐year‐old population had not yet reached reproductive maturity, and both plant and seedling densities were low (Marteinsdóttir et al., 2007). In the full dataset from 2008 (6000 m^2^), no first‐year seedlings and only 25 S‐seedlings (6% of sample) were recorded. However, in 2018, S‐seedlings were estimated to be over 6000 (83% of the sample), and first‐year seedlings were in the thousands. As we did not detect exceptional weather events or growing season trends through the study period (unpublished data from the Icelandic Meteorological Office, www.vedur.is), we propose that the recent surge in the seedling establishment is linked to the previously described surge in seed production, indicating greater limitation by seed than microsite early on in our study. If most of the newly established seedlings have a local origin, then this signals a turning point in the population's development since the persistence of a population requires that colonizers leave descendants in the new range (Hargreaves & Eckert, 2014). However, for a lasting impact, the long‐term survival of those seedlings is needed. If some of these recruits persist, then the time from the initial colonization in ca. 1990 to the establishment of the first locally recruited generation can be estimated to be of the order of 25 years. It is certainly more than 15 years, and less than 30 years.

### 4.2 Emerging spatial and temporal patterns

Most of the population variables showed significant variation among sites and years, and several also had *year x site* interactions. Sites S3 and S4 appeared quite alike in 2008, having similar growth form indices, and S3 had a slightly (although not statistically significant) greater mean tree size and density of plants, trees, flowering trees, and catkins (Table 2). By 2018, this had been reversed. Then, the 20 largest trees at S4 were significantly larger than at S3, plants had a significantly higher growth form index and five times greater (although not statistically different) density of catkins (Figures 5 and 6b, Table 2). Sites S1 and S2 are more difficult to place with respect to S3 and S4. Although our unpublished data (H. M. Birkisdóttir et al.) do not indicate that their oldest birch plants are younger, they were smaller and sparser in 2008, had a smaller increase in average tree size between years, and had lower catkin densities in 2018 (Table 2, Figures 4 and 5). An intriguing anomaly is that while the growth form index increased from 2008–2018 at S1 and S4, indicating a shift to more upright growth, it actually decreased at S2 and S3, with plants becoming more decumbent (Figure 6a). Roughly, the sites fall into two classes. S4 has largely monocormic trees with a high growth rate, high fecundity, and extremely high second‐generation seedling densities. Two of the other sites (S2 and S3) have largely decumbent shrubby birch with lower growth rates, much lower catkin densities, and very limited second‐generation recruitment. S1 partly resembles S2 and S3, but its shift in growth form index, increase in the size of the 20 largest trees, and its 2018 values of density of plants, trees, and flowering plants in comparison with S4 values in 2008 may indicate that it may fall more in line with S4, but with a time lag.

The two populations within Vatnajökull National Park are quite different. The young VM population had a similar average tree size and growth form as S4 in 2018 (Figures 4b and 6b), but the very low percentage of flowering at VM precludes comparison of reproductive traits. Meanwhile, the older VS population had very different traits. It had by far the highest tree density and the greatest density of flowering plants (Table 2), but in many other respects, it falls in line with S3. VS plants had the most decumbent growth form of all the populations, trees were significantly smaller than at S4 and VM, and they had the lowest number of catkins relative to size (Figures 3b, 4b, and 6b). Our results are in line with Thórsson et al. (2007), who contrasted the procumbent and shrubby plants in Skaftafell (VS) with the tall monocormic trees in the old forest in Bæjarstaðarskógur, close to VM. (Figure 6b). Mountain birch has a notoriously variable growth form, ranging from decumbent (sometimes virtually horizontal) polycormic shrubs to monocormic upright trees that in Iceland may reach 10–12 m. The shrubby form is typical of highly oceanic, windy, and higher‐elevation sites, with the tree form dominating in more benign locations, e.g., lowland valleys (Atkinson, 1992; Jónsson, 2004; Verwijst, 1988). This structural diversity has both been attributed to high phenotypic plasticity and to hybridisation with *Betula nana* (Thórsson et al., 2007; Verwijst, 1988).

For trees in general, greater physiological plasticity has been associated with shade‐intolerant species colonizing early successional habitats (Portsmuth & Niinemets, 2007) but the plastic responses of tree architecture to local environmental conditions are generally not well known (Van de Peer et al., 2017). Neither has been investigated for *Betula pubescens*. Skeiðarársandur and vicinities have a milder climate and longer growing season than most of the rest of Iceland, and the old forest at Bæjarstaðarskógur harbors some of Iceland's tallest mountain birches. The study sites are, therefore, climatically well within the range occupied by the monocormic tree form. Jónsson (2004) concluded that the growth form variation in Icelandic mountain birch is accompanied by differences in growth rates, with the upright monocormic form having faster growth than the shrubby decumbent form. Positive correlation between growth rate and life expectancy has also been found (Jónsson, 2004), indicating that greater canopy height and stand age might be expected at VM and at least in parts of Skeiðarársandur than at VS.

In mountain birch, age/size at reproductive maturity varies among individuals and reflects environmental conditions (Aradóttir, 1991; Atkinson, 1992). Here, the predicted probability of catkin presence increased rapidly at all sites from a threshold plant size of ca. 50 cm (Figure 3a; VM excluded due to low flowering frequency). For catkin abundance per flowering plant, the difference between VS and S4 was especially apparent (Figure 3b), and although the density of flowering plants was more than three times greater at VS than S4, catkin density at VS was less than a quarter of the density at S4 (Table 2).

The number of flowers produced is generally positively correlated with plant size (Fenner & Thompson, 2005; Table 3), but this is unlikely to fully explain the difference in catkin abundance between S4 and VS (Figure 3b). Resources may also limit reproduction (Campbell & Halama, 1993). Soil carbon and nitrogen data (%) are available for S4 (J. B. U. Tómasson et al. unpublished data), and VS's close vicinity (Vilmundardóttir et al., 2015). All samples were very low in soil fertility, but it was slightly higher near VS in 2010–2011 (*C* = 1.77 ± 1.10, *N* = 0.101 ± 0.064 [means ± standard deviation], *n* = 18) than at S4 in 2018 (*C* = 1.42 ± 0.92, *N* = 0.044 ± 0.015, *n* = 16). Soil nutrients are therefore unlikely to explain the difference in catkin production of flowering trees between the two sites in 2018, and to elucidate the reproduction dynamics of the young population, more research is needed on temporal variation in flowering and possible explanations.

### 4.3 Site divergence and possible environmental correlates

Two hypotheses may be advanced to explain the divergence across Skeiðarársandur, first that despite the highly similar climate and apparent homogeneity of the plain, there is sufficient environmental heterogeneity to induce the observed difference in growth and dynamics, and second that the population differs genetically among sites. We begin by considering the first hypothesis.

With time, the young mountain birch population on Skeiðarársandur has developed diverging patterns among sites (Tables 1, 4, and 5), roughly dividing them into two classes by 2018 (see Section 4.2). Since the temporal divergence was particularly noticeable for S3 and S4, we focus our discussion on those sites. In 2008, the two could not be distinguished in terms of plant densities or size distributions and were predicted to advance at comparable rates (Hiedl et al., 2009). Unexpectedly, this turned out not to be the case, resulting in them being characterized into different classes by 2018. Sites S3 and S4 are only 500 m apart and appear very alike to the human eye. One noticeable difference between them was the unequal increase in plant density (Table 2), largely explained by the huge number of seedlings at S4 in 2018.

Seedling establishment is one of the most crucial stages in a plant's life cycle and can be affected by a range of factors, including microhabitat (Lett et al., 2017; Nystuen et al., 2019), herbivores (Speed et al., 2010), soil moisture (Pinto et al., 2016), nutrient status (Harper, 1977), mycorrhizal associations (Kokkoris et al., 2020), and diverse combinations of interactions among factors (Lett & Dorrepaal, 2018). Preliminary results from vegetation analyses at the study sites (G. Óskarsdóttir et al., unpublished data) suggest that the sward layer at S3 and S4 is similar enough for microsite limitations to be comparable (see Section 2.2). *Racomitrium* mosses dominated the sward layer vegetation, and their average thickness was 1.3 cm at both sites. Effect of mosses on the seedling establishment is dependent on their traits and varies with climate (Lett et al., 2017). In Iceland, thin moss (<2 cm) has been shown to constitute a favorable microsite for mountain birch establishment (Aradóttir & Halldórsson, 2018). Thus, we conclude that the relatively few seedlings at S3 cannot be ascribed to the scarcity of microsites.

Another potential check on the seedling establishment is herbivory (Speed et al., 2010; Thórsson, 2008). Skeiðarársandur is not protected from grazing, but during summer, only 200 ewes graze the vast but mostly sparsely vegetated plain (Thórhallsdóttir & Svavarsdóttir, 2022). At our sites, low frequency of grazing marks attributed to sheep, recorded on 8% and 1% of mountain birch plants (S‐seedlings excluded) in 2008 and 2018, respectively (G. Óskarsdóttir et al., unpublished data), suggests limited impact on the population. Furthermore, given the short distance and absence of barriers between S3 and S4, it is scarcely conceivable that the difference can be assigned to grazing.

The lack of obvious above‐ground environmental differences between the two sites raises questions on possible variation in soil properties. Plant growth is often resource‐limited (Ågren et al., 2012), especially in early succession (Marteinsdóttir et al., 2018; Vitousek et al., 1993). At both sites, trees were significantly larger in 2018 than in 2008, and within years, tree size was not statistically different between sites (Figure 4). However, spatial divergence in size of the largest trees (Figure 5) suggests that conditions for growth had indeed been more favorable at S4 than S3 in the study period, and resources may have been more limiting at S3 than S4. Comparison of soil properties between sites are needed to clarify this.

On Skeiðarársandur, the establishment of mountain birch is likely to increase rates of ecosystem development, e.g., by improving soil physical properties and nutrient status (Jonczak et al., 2020; Weidlich et al., 2020), increasing litter production and organic matter accumulation (McElhinny et al., 2010), ameliorating microclimate (D'Odorico et al., 2013), and changing above‐ and below‐ground communities and successional processes (Kittipalawattanapol et al., 2021; Mitchell et al., 2007, 2010; Quinn et al., 2021). Consequently, the mountain birch population can leave a long‐term legacy that steers the community's successional pathway for years and decades to come (García‐Girón et al., 2022).

### 4.4 Historical contingency and population development

The question of how the assembly of biological communities is influenced by past history remains a core issue in ecology (Chase, 2003; Fukami et al., 2016). Among key concepts are legacies or ecological memories, which have especially been explored in relation to disturbances and forest ecosystems (Johnstone et al., 2016), and in a sense, ecosystem succession can at least sometimes be considered as an expression of biological legacy. We contend that these issues also need to be considered at the intraspecific level, and that this may be particularly relevant for arctic and subarctic ecosystems. While the arctic/subarctic vascular flora is species poor compared to most other biomes (Grundt et al., 2006; Väre et al., 2013), it is now recognized that this may mask ecologically important but largely cryptic variation at the subspecies level (e.g., Brochmann & Brysting, 2008; Steltzer et al., 2008; Stubbs et al., 2020). Furthermore, these issues are likely to be particularly relevant in early succession, for example, when a tree species establishes in a community composed of low‐growing vegetation. This qualifies as a priority effect in the sense of niche modification, as defined by Fukami (2015).

As already discussed (Section 4.3), a spatial divergence of the Skeiðarársandur population is evident in almost all the population variables recorded: plant growth form, tree size, and catkin as well as seedling densities (Figures 4 and 6, Table 2). There are two possible explanations for that spatial divergence. The first is that despite the apparent homogeneity of Skeiðarársandur outwash plain, there was sufficient underlying heterogeneity in the substrate at the site scale to significantly affect the aboveground structure (see Section 4.3). The millennial build‐up of Skeiðarársandur is largely due to frequent and massive glacial outburst floods, with the largest Little Ice Age floods extending over more or less the entire 1000 km^2^ plain (Thórhallsdóttir & Svavarsdóttir, 2022). While there is a well recognizable seawards gradient in soil grain size, it seems rather unlikely that soil properties can differ sufficiently in the short distance (500 m) between S3 and S4, to account for the demographical differences. The second explanation is that the birch on Skeiðarársandur has originated from genetically different sources. At this moment, we are unable to distinguish between the two.

Irrespective of the nature of the variation, we conclude that the young mountain birch population on Skeiðarársandur appears to be set on diverging trajectories. On one hand, there are the fast‐growing, largely monocormic plants with massive recruitment of second‐generation seedlings at S4, and on the other, the slower growing, polycormic plants with limited recruitment, most pronounced at S2. While acknowledging that the role of soil heterogeneity is unresolved, we suggest that the large‐scale establishment of mountain birch on Skeiðarársandur is an example of how the stochastic colonization of a niche‐modifying species is set to leave an ecosystem impact with a significantly different intraspecific imprint and a long‐term legacy. This is all the more remarkable in light of the apparent homogeneity of the flat and featureless outwash plain environment.

In the context of distribution shifts due to global climate change, our study may provide lessons. Although directly (climate warming) and indirectly (glacier retreat) mediated by climate change, the colonization of mountain birch on Skeiðarársandur was a natural process (Thórhallsdóttir & Svavarsdóttir, 2022). The massive long‐distance (≥10 km) dispersal, spatially extensive colonization (>35 km^2^ area from ca. 1990–2016), and high, although spatially variable, recruitment of the second generation, all illustrate the mountain birch's ability to rapidly adjust its range in a shifting environment.

## AUTHOR CONTRIBUTIONS

**Guðrún Óskarsdóttir:** Formal analysis (equal); investigation (equal); visualization (lead); writing – original draft (lead); writing – review and editing (lead). **Thora Ellen Thorhalsdottir:** Conceptualization (equal); data curation (equal); funding acquisition (equal); investigation (equal); methodology (equal); supervision (equal); visualization (supporting); writing – original draft (lead); writing – review and editing (lead). **Anna Helga Jónsdóttir:** Formal analysis (equal); visualization (supporting); writing – original draft (supporting); writing – review and editing (supporting). **Hulda Margrét Birkisdóttir:** Investigation (equal); writing – original draft (supporting); writing – review and editing (supporting). **Kristín Svavarsdóttir:** Conceptualization (equal); data curation (equal); funding acquisition (equal); investigation (equal); methodology (equal); supervision (equal); visualization (supporting); writing – original draft (lead); writing – review and editing (lead).

## CONFLICT OF INTEREST

The authors have no conflict of interest to declare.

## Supporting information


Tables S1–S4

## Data Availability

Data are available in the Dryad public repository: https://doi.org/10.5061/dryad.4b8gthtgj.
