# Supplementary material for: Establishment of mountain birch (Betula pubescens ssp. tortuosa) on a glacial outwash plain: Spatial patterns and decadal processes
Source: Ecol Evol. 2022 Oct 27;12(10):e9430. doi: 10.1002/ece3.9430 (PMC9608789; doi:10.1002/ece3.9430)
Supplement: Supplementary file 1 — Tables S1–S4 [file ECE3-12-e9430-s001.docx]

TABLE S1. Summary of models run.

| **Model ID** | **Model description** | **Model equation** | **Data** | **R-code** |
| --- | --- | --- | --- | --- |
| 1Ai | Negative binomial mixed model | n(All plants per transect) ~ Year x Site + (1\|Site/Transect) | SKS 2008 and 2018 | glmmTMB (family = nbinom2 (link = ”log”) |
| 1Aii | Negative binomial mixed model | n(Trees per transect) ~ Year x Site + (1\|Site/Transect) | SKS 2008 and 2018 | glmmTMB (family = nbinom2 (link = ”log”) |
| 1Aiii | Negative binomial mixed model | n(Flowering adults per transect) ~ Year x Site+ (1\|Site/Transect) | SKS 2008 and 2018 | glmmTMB (family = nbinom2 (link = ”log”) |
| 1Aiv | Negative binomial mixed model | n(Catkins per transect) ~ Year x Site + (1\|Site/Transect) | SKS 2008 and 2018 | glmmTMB (family = nbinom2 (link = ”log”) |
| 1Bi | Negative binomial model | n(All plants per transect) ~ Site | SKS 2008 | glm.nb |
| 1Bii | Negative binomial model | n(Trees per transect) ~ Site | SKS 2008 | glm.nb |
| 1Biii | Negative binomial model | n(Flowering adults per transect) ~ Site | SKS 2008 | glm.nb |
| 1Biv | Negative binomial model | n(Catkins per transect) ~ Site | SKS 2008 | glm.nb |
| 1Ci | Negative binomial model | n(All plants per transect) ~ Site | SKS and VNP 2018 | glm.nb |
| 1Cii | Negative binomial model | n(Trees per transect) ~ Site | SKS and VNP 2018 | glm.nb |
| 1Ciii | Negative binomial model | n(Flowering adults per transect) ~ Site | SKS and VNP 2018 | glm.nb |
| 1Civ | Negative binomial model | n(Catkins per transect) ~ Site | SKS and VNP 2018 | glm.nb |
| 2Ai | Hurdle/zero-altered negative binomial mixed models | Catkins ~ Site + Size x Year + (1\|Site/Transect), zi = ~ Site + Size x Year | SKS 2008 and 2018, S- and L-seedlings excluded | glmmTMB (family = truncated_nbinom2 (link = “log")) |
| 2Aii | Hurdle/zero-altered negative binomial mixed models | Catkins ~ Size + (1\|Site/Transect), zi = ~ Site + Size + Year | SKS 2008 and 2018, S- and L-seedlings excluded | glmmTMB (family = truncated_nbinom2 (link = “log")) |
| 2Bi | Hurdle/zero-altered negative binomial mixed models | Catkins ~ Site x Size + (1\|Site/Transect), zi = ~ Site x Size | SKS and VNP 2018, S- and L-seedlings excluded | glmmTMB (family = truncated_nbinom2 (link = “log")) |
| 2Bii | Hurdle/z-altered negative binomial mixed models | Catkins ~ Site + Size + (1\|Site/Transect), zi = ~ Site + Size | SKS and VNP 2018, S- and L-seedlings excluded | glmmTMB (family = truncated_nbinom2 (link = “log")) |
| 3Ai | Linear mixed model | log(Size) ~ Year x Site, random = ~ 1\|Site/Transect, weights = varIdent(form = ~ 1\|Year) | SKS 2008 and 2018, trees | lme (method = "REML") |
| 3Aii | Linear model | log(Size) ~ Year x Site | SKS 2008 and 2018, 20 largest trees each site | lm |
| 3Bi | Linear mixed model | log(Size) ~ Site, random = ~ 1\|Site/Transect | SKS 2008 | lme (method = "REML") |
| 3Bii | Linear model | log(Size) ~ Site | SKS 2008, 20 largest trees each site | lm |
| 3Ci | Linear mixed model | log(Size) ~ Site, random = ~ 1\|Site/Transect | SKS and VNP 2018 | lme (method = "REML") |
| 3Cii | Linear model | Size ~ Site | SKS and VNP 2018, 20 largest trees each site | lm |
| 4A | Linear mixed model | Size/Height ~ Year x Site, random = ~ 1\|Site/Transect, weights = varIdent(form = ~ 1\|Year) | SKS 2008 and 2018, S- and L-seedlings excluded | lme (method = "REML") |
| 4B | Linear mixed model | Size/Height ~ Site, random = ~ 1\|Site/Transect | SKS 2008, S- and L-seedlings excluded | lme (method = "REML") |
| 4C | Linear mixed model | Size/Height ~ Site, random = ~ 1\|Site/Transect | SKS and VNP 2018, S- and L-seedlings excluded | lme (method = "REML") |

*Note*: Trees = ≥20 cm plants, flowering adults = plants with female catkins, S-seedlings = ≤1 cm plants, L-seedlings = 1–5 cm plants. Since all transect were the same size (150 m^2^), spatial and temporal variation in plant/catkin density (models 1Ai to 1Civ) was estimated using negative binomial regression on the number of plants/catkins. For models 2Aii and 2Bii, equations represent the best subset models for 2Ai and 2Bi, respectively, according to backwards elimination using a likelihood ratio test (see results in Table S3).

TABLE S2. Pairwise temporal comparisons for each site in models 1Ai to 1Aiv in Table S1, according to estimated marginal means.

| **Model ID** | **Contrasts** | **Estimate** | **SE** | ***t*-value** | ***p*** |
| --- | --- | --- | --- | --- | --- |
| 1Ai | S1 (2008–2018) | -0.940 | 0.276 | -3.406 | **0.001** |
|  | S2 (2008–2018) | -1.329 | 0.356 | -3.729 | **0.005** |
|  | S3 (2008–2018) | -0.221 | 0.196 | -1.131 | 0.264 |
|  | S4 (2008–2018) | -4.646 | 0.285 | -16.296 | **<0.001** |
| 1Aii | S1 (2008–2018) | -1.130 | 0.353 | -3.206 | **0.002** |
|  | S2 (2008–2018) | -0.145 | 0.484 | -0.299 | 0.766 |
|  | S3 (2008–2018) | -0.116 | 0.183 | -0.635 | 0.528 |
|  | S4 (2008–2018) | -0.734 | 0.276 | -2.655 | **0.011** |
| 1Aiii | S1 (2008–2018) | -2.835 | 1.034 | -2.741 | **0.009** |
|  | S2 (2008–2018) | -1.943 | 1.076 | -1.805 | 0.077 |
|  | S3 (2008–2018) | -0.965 | 0.243 | -3.979 | **<0.001** |
|  | S4 (2008–2018) | -1.514 | 0.393 | -3.848 | **<0.001** |
| 1Aiv | S1 (2008–2018) | -5.100 | 1.207 | -4.226 | **<0.001** |
|  | S2 (2008–2018) | -2.790 | 1.001 | -2.788 | **0.008** |
|  | S3 (2008–2018) | -1.100 | 0.626 | -1.755 | 0.086 |
|  | S4 (2008–2018) | -2.210 | 1.044 | -2.120 | **0.039** |

*Note*: Significant values are in bold (*p*<0.05).

TABLE S3. Results of model selection for models 2Ai and 2Bi in Table S1, according to backwards elimination using a likelihood ratio test.

| **Model ID** | **Part of model** | **Dropped term** | **AIC** | **Likelihood ratio test** | |
| --- | --- | --- | --- | --- | --- |
|  |  |  |  | ***χ^2^*** | ***p*** |
| 2Ai |  | None | 2098.5 |  |  |
|  | zero | Year x Plant size | 2098.0 | 1.498 | 0.221 |
|  | zero | Year | 2104.4 | 8.419 | **0.004** |
|  | zero | Site | 2105.9 | 13.947 | **0.003** |
|  | zero | Plant size | 2417.7 | 321.69 | **<0.001** |
|  | count | Year x Plant size | 2098.4 | 2.431 | 0.119 |
|  | count | Year | 2097.1 | 0.694 | 0.405 |
|  | count | Site | 2098.2 | 7.089 | 0.069 |
|  | count | Plant size | 2132.6 | 36.433 | **<0.001** |
| 2Bi |  | None | 2446.0 |  |  |
|  | zero | Site x Plant size | 2444.8 | 6.813 | 0.146 |
|  | zero | Site | 2457.6 | 20.794 | **<0.001** |
|  | zero | Plant size | 2815.4 | 372.64 | **<0.001** |
|  | count | Site x Plant size | 2438.5 | 1.655 | 0.799 |
|  | count | Site | 2444.2 | 13.761 | **0.008** |
|  | count | Plant size | 2469.2 | 32.761 | **<0.001** |

*Note*: Significant values are in bold (*p*<0.05).

TABLE S4. All pairwise comparisons for models 2Aii and 2Bii in Table S1, according to estimated marginal means.

| **Model ID** | **Contrasts** | **Estimate** | **SE** | ***t*-value** | ***p*** |
| --- | --- | --- | --- | --- | --- |
| 2Aii (zero-part) | S1 – S2 | 0.736 | 0.580 | 1.268 | 0.584 |
|  | S1 – S3 | 0.228 | 0.389 | 0.585 | 0.937 |
|  | S1 – S4 | -0.829 | 0.449 | -1.849 | 0.252 |
|  | S2 – S3 | -0.508 | 0.500 | -1.017 | 0.739 |
|  | S2 – S4 | -1.565 | 0.553 | -2.829 | **0.025** |
|  | S3 – S4 | -1.057 | 0.325 | -3.250 | **0.007** |
|  | 2008 – 2018 | 0.752 | 0.263 | 2.855 | **0.004** |
| 2Bii (zero-part) | S1 – S2 | 0.748 | 0.630 | 1.189 | 0.758 |
|  | S1 – S3 | 0.310 | 0.411 | 0.755 | 0.943 |
|  | S1 – S4 | -1.178 | 0.475 | -2.479 | 0.097 |
|  | S1 – VS | -0.170 | 0.391 | -0.434 | 0.993 |
|  | S2 – S3 | -0.438 | 0.559 | -0.784 | 0.935 |
|  | S2 – S4 | -1.927 | 0.612 | -3.151 | **0.015** |
|  | S2 – VS | -0.918 | 0.541 | -1.697 | 0.436 |
|  | S3 – S4 | -1.489 | 0.368 | -4.044 | **<0.001** |
|  | S3 – VS | -0.480 | 0.259 | -1.855 | 0.343 |
|  | S4 – VS | 1.009 | 0.354 | 2.850 | **0.036** |
| 2Bii (count-part) | S1 – S2 | -0.965 | 0.792 | -1.219 | 0.740 |
|  | S1 – S3 | -1.083 | 0.478 | -2.265 | 0.157 |
|  | S1 – S4 | -1.635 | 0.549 | -2.980 | **0.025** |
|  | S1 – VS | 0.100 | 0.493 | 0.204 | 1.000 |
|  | S2 – S3 | -0.118 | 0.705 | -0.167 | 1.000 |
|  | S2 – S4 | -0.670 | 0.771 | -0.869 | 0.908 |
|  | S2 – VS | 1.066 | 0.718 | 1.483 | 0.574 |
|  | S3 – S4 | -0.552 | 0.418 | -1.319 | 0.679 |
|  | S3 – VS | 1.184 | 0.349 | 3.388 | **0.007** |
|  | S4 – VS | 1.736 | 0.438 | 3.963 | **0.001** |

*Note*: For model 2Aii, pairwise comparisons were only performed for the zero-part, as neither site nor year were significant in the count-part. Significant values are in bold (*p*<0.05).
